# WW Domain-Containing Proteins YAP and TAZ in the Hippo Pathway as Key Regulators in Stemness Maintenance, Tissue Homeostasis, and Tumorigenesis

**DOI:** 10.3389/fonc.2019.00060

**Published:** 2019-02-11

**Authors:** Yu-An Chen, Chen-Yu Lu, Tian-You Cheng, Szu-Hua Pan, Hsin-Fu Chen, Nan-Shan Chang

**Affiliations:** ^1^Graduate Institute of Medical Genomics and Proteomics, College of Medicine, National Taiwan University, Taipei, Taiwan; ^2^Department of Optics and Photonics, National Central University, Chungli, Taiwan; ^3^Department of Obstetrics and Gynecology, College of Medicine and the Hospital, National Taiwan University, Taipei, Taiwan; ^4^Institute of Molecular Medicine, College of Medicine, National Cheng Kung University, Tainan, Taiwan; ^5^Department of Neurochemistry, New York State Institute for Basic Research in Developmental Disabilities, New York, NY, United States; ^6^Graduate Institute of Biomedical Sciences, College of Medicine, China Medical University, Taichung, Taiwan

**Keywords:** Hippo pathway, WW domain proteins, tissue homeostasis, regeneration, stem cell, induced pluripotent stem cells, tumorigenesis

## Abstract

The Hippo pathway is a conserved signaling pathway originally defined in *Drosophila melanogaster* two decades ago. Deregulation of the Hippo pathway leads to significant overgrowth in phenotypes and ultimately initiation of tumorigenesis in various tissues. The major WW domain proteins in the Hippo pathway are YAP and TAZ, which regulate embryonic development, organ growth, tissue regeneration, stem cell pluripotency, and tumorigenesis. Recent reports reveal the novel roles of YAP/TAZ in establishing the precise balance of stem cell niches, promoting the production of induced pluripotent stem cells (iPSCs), and provoking signals for regeneration and cancer initiation. Activation of YAP/TAZ, for example, results in the expansion of progenitor cells, which promotes regeneration after tissue damage. YAP is highly expressed in self-renewing pluripotent stem cells. Overexpression of YAP halts stem cell differentiation and yet maintains the inherent stem cell properties. A success in reprograming iPSCs by the transfection of cells with Oct3/4, Sox2, and Yap expression constructs has recently been shown. In this review, we update the current knowledge and the latest progress in the WW domain proteins of the Hippo pathway in relevance to stem cell biology, and provide a thorough understanding in the tissue homeostasis and identification of potential targets to block tumor development. We also provide the regulatory role of tumor suppressor WWOX in the upstream of TGF-β, Hyal-2, and Wnt signaling that cross talks with the Hippo pathway.

## Introduction

The Hippo pathway was originally identified by genetic screens of tumor suppressors for tissue growth control in *Drosophila melanogaster*. Recent advances in the identification of the mammalian Hippo pathway components and functional implications highlight the role of the pathway in organ development, tissue regeneration, stem cell maintenance, and tumorigenesis (1–4). The pathway is evolutionarily conserved.

The stem cell has indeed generated a great interest for scientists in the past decades and attracted more and more attention from the public recently. The unique properties of stem cells give substantial clues for clinical doctors, biologists, and scientists to solve problems in fundamental biology during the developmental process and the aberrant progression of degenerative diseases and cancer. Despite extensive investigations into the underlying mechanisms of stemness properties, what has not been known is the key signaling pathway, which orchestrates the network for conferring stemness maintenance and tissue homeostasis. Exploration of the Hippo pathway on stem cell biology has shed light on the developmental path (5–8). Here, we focus on discussing how the key WW domain-containing YAP and TAZ of the Hippo pathway contribute their regulatory role via the WW domain and PY (proline-tyrosine) motif interactions.

## The Hippo Pathway

The Hippo pathway took the name from aberrant tissue overgrowth and neoplasia that generates a unique “hippopotamus”-like phenotype. Beginning in 1995, two studies first discovered the Warts (Wts) gene deletion, which caused robust multiple tissue overgrowth in *D. melanogaster* (9, 10). Later, researchers uncovered more components within this pathway, including scaffolding protein Salvador (Sav) (11), Ste20-like kinase Hippo (Hpo) (12–14), and Mob as tumor suppressor (Mats) (15). These mutant proteins may cause tissue overgrowth in *Drosophila*. Soon after the discovery of the Salvador/Warts/Hippo pathway, Yorkie (Yki) was shown to be one of the key functional effectors of this pathway from the screening of Wts-interacting proteins (16). The Hippo pathway-regulated overgrowth in organs has quickly attracted broad attention and led to the development of transgenic mouse models (16, 17).

### Core Components of Hippo Pathway

**Figure 1:**
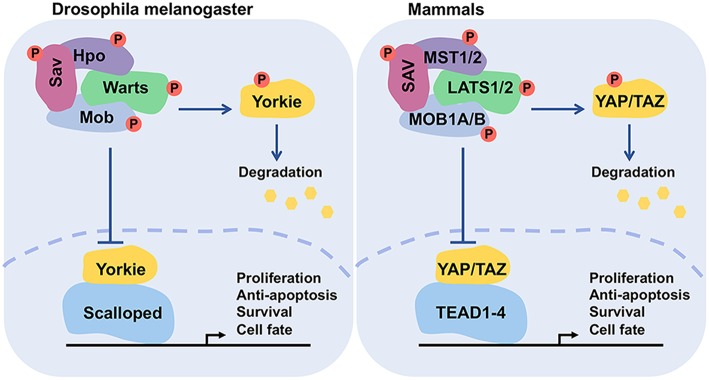
Functional conservation of the core components of Hippo pathway. The functionally conserved factors of *Drosophila melanogaster* and mammals are matched by color. This network controls the transcriptional events for regulating cell proliferation, survival, and death.

The Hippo pathway is essentially a cascade of kinases, comprising transcription coactivators and DNA-binding proteins. Although the pathway was first discovered in *Drosophila*, it is evolutionarily conserved in mammals. The mammalian orthologs of Hpo, Sav, Wts, Mats, and Yki are Mammalian sterile 20-like 1/2 (MST1/2, also named as STK4/3), Salvador (SAV1), Large tumor suppressor homolog 1/2 (LATS1/2), MOB kinase activator 1A/B (MOB1A/B), and Yes-associated protein (YAP)/Transcriptional co-activator with PDZ binding motif (TAZ), respectively (Figure 1 and Table 1) (15, 18–21, 29). The best-known role of the Hippo pathway is to orchestrate organ development and control tissue homeostasis through modulation of cell proliferation, apoptosis, migration, and differentiation (7, 8). Hippo pathway also regulates stem cell self-renewal and expansion and tissue regeneration (30–32).

**Table 1:** Hippo pathway components and major functions.

**Drosophila**	**Mammals**	**Major function in Hippo pathway**	**References**
Hippo (Hpo)	MST1 MST2	Phosphorylate LAST1/2, MOB1, and SAV, leading to LAST1/2 activation	(18)
Salvador (Sav)	SAV1	Interact with MST1/2, promotes phosphorylation of LAST1/2 by MST1/2	(19)
Warts (Wts)	LATS1 LATS2	Phosphorylate and inactivate YAP/TAZ	(20)
Mats	MOB1A/B	Scaffold protein of LAST1/2	(15)
Yorkie (Yki)	YAP TAZ	Transcription co-activator, major effectors of the Hippo pathway	(21)
Scalloped (Sd)	TEAD1-4	Transcription factors mediate the effect of YAP/TAZ	(21)
Tgi	VGLL4	Competes with YAP/TAZ for TEADs, inhibits YAP/TAZ functions	(22)
Misshapen (Msn)	MAP4K4	May form a complex and mediates upstream signals. (from plasma membrane) to MST1/2. Activates LAST1/2	(23)
Merlin (Mer)	NF2	Signal transduction between cytoskeletal proteins and cell membrane. May bring LAST1/2 to plasma membrane and facilitate its activation by MST1/2.	(24)
Kibra	KIBRA	Phosphoprotein involved in cell polarity, mitosis and cell migration	(25)
Expanded (Ex)	FRMD6 AMOT RASSF1	Protein linking cytoskeleton to plasma membrane Sequesters YAP/TAZ to cell junctions, binding and indirectly activates LAST1/2, a substrate of LAST1/2 Signal transducer, microtubule stabilization, cell cycle arrest	(26) (27) (28)

### Numerous Initiating Signals for the Responsive Kinase Cascade: An Integrated Action or a Conundrum in Chaos?

**Figure 2:**
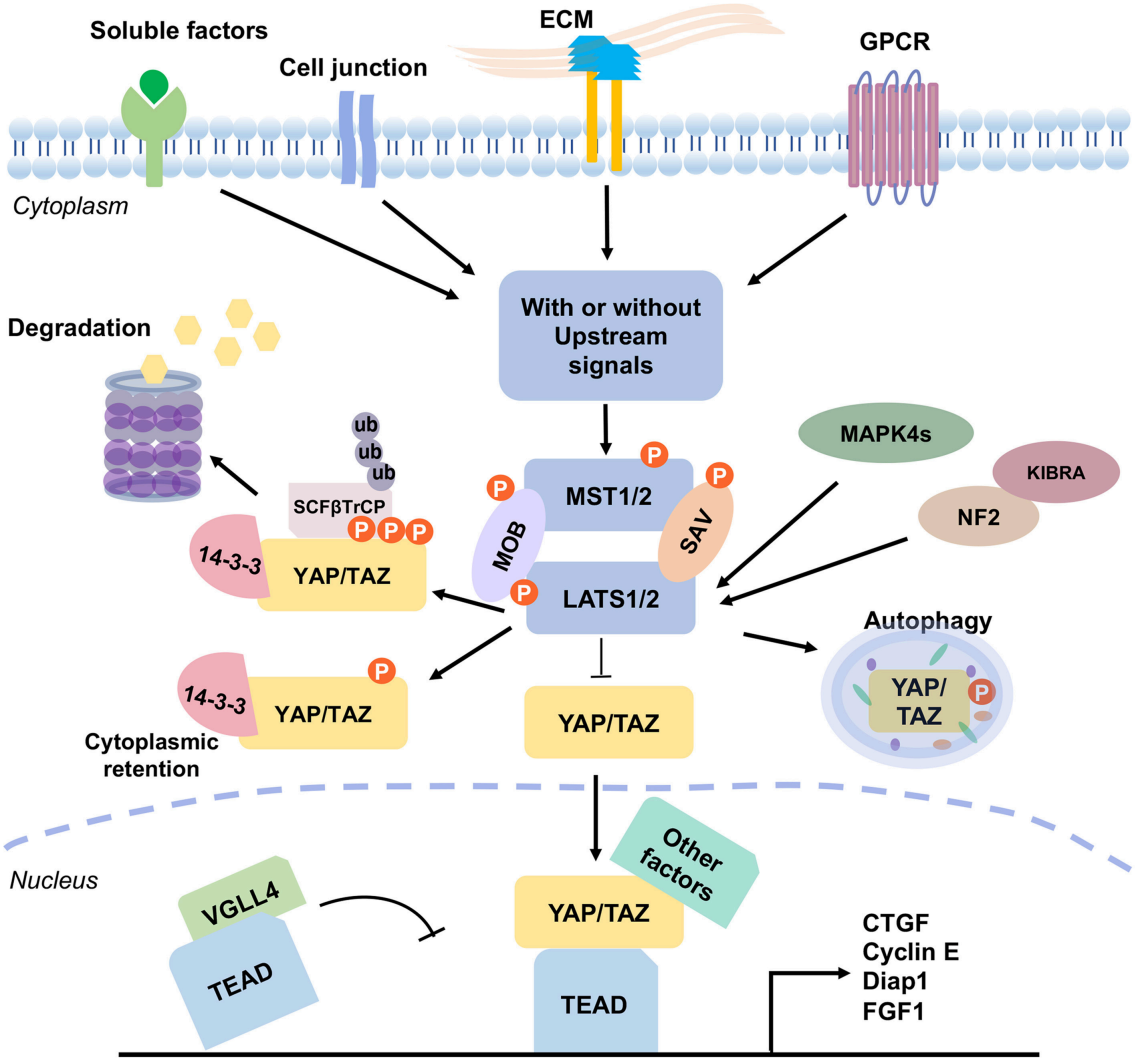
The mammalian Hippo pathway signaling. Various signal stimulators activate the Hippo pathway, which includes GPCR, cell junction and ECM proteins, and soluble factors. These signals regulate the phosphorylation of MST1/2 and LATS1/2 kinases to further induce YAP/TAZ activation. Without the upstream signals, KIBRA interacts with NF2 to promote the pathway activity. Also, NF2 and MAPK4s directly interact with LAST1/2 and promote LATS1/2 phosphorylation (pLATS1/2). Then, pLATS1/2 phosphorylates YAP/TAZ. And, the phosphorylated YAP/TAZ presents a 14-3-3 biding site and SCFβTrCP specific recognition site, which causes cytoplasmic retention of YAP/TAZ and results in YAP/TAZ degradation. YAP protein is also degraded via autophagy. VGLL4 acts as a transcriptional repressor by competing with YAP/TAZ for binding TEADs. Unphosphorylated YAP/TAZ complex translocates into the nucleus, and acts as transcription coactivators by forming complexes with various transcriptional factors, such as TEADs. Binding of YAP/TAZ with TEAD prevents VGLL4 from associating with the complex, and consequently drives the expression of numerous genes which are related to cell proliferation, survival, and migration. The signaling outcome includes induction of cell cycle regulator cyclin E, cell-death inhibitor Diap1, connective tissue growth factor (CTGF), and fibroblast growth factor (FGF1), and etc.

The Hippo pathway receives a broad range of signals, including extracellular mechanical force, hormonal cues, and intrinsic cell physical mechanism, so as to control cell polarity and cytoskeletal organization, and interactions with proteins in the extracellular matrix (33–35). Numerous proteins participate in the initiation of the Hippo signaling. For example, protein kinases, including Src kinase, protein kinase A (PKA), PAR-1/MARK kinase, and TAO kinase are involved (1, 36). Extracellular soluble factor Amphiregulin (37), matrix Agrin proteoglycan and Integrins (38), and cell junctional proteins Echinoid and Cadherin-Catenin complex (39, 40) participate in the signaling network. Furthermore, cell polarity proteins Crumbs, and Scribble complexes (41, 42), G-protein-coupled receptor (GPCR) and Wnt/beta-catenin signal pathways (43, 44), and cytoskeletal Spectrin, Myosin II, and F-actin (45–49) are integrated in the signaling network (Figure 2). Among these signals, many of them collaborate with each other, whereas others may be in conflict. How each cell selectively sorts out which signal(s) to respond and/or converge all signals, or ignore and terminate them, remains to be a critical issue to resolve.

When cells receive the extracellular upstream activation signals, the signal receiver MST1/2 (or *Drosophila* Hpo) phosphorylates LATS1/2 (or *Drosophila* Wts) and MOB1 (or *Drosophila* Mats) in a canonical manner, with the assistance of cofactor SAV1 (or *Drosophila* Sav). SAV1 is a WW domain-containing protein needed for integrating the upstream signal(s). Then, the activated LATS1/2, in turn, triggers the phosphorylation of the major coactivators YAP/TAZ (two homologs of *Drosophila* Yki) at multiple residues (Figure 1). Phosphorylation of YAP at S127 (corresponding to S89 on TAZ) promotes its binding with 14-3-3, thus resulting in the cytoplasmic retention (20). Phosphorylation of YAP/TAZ at S381 and S311, respectively, creates a binding site for casein kinase 1 (CK1) and subsequent phosphorylation by CK1δ/ε at the DSGxS motif. Then SCFβTrCP, a multi-subunit SKP-CULLIN-F-box (SCF) ligase complex specifically recognizes the phosphodegron DpSGxpS of YAP and TAZ for leading to eventual YAP/TAZ ubiquitination and degradation (20, 50, 51). YAP protein is also degraded via autophagy (52). Unphosphorylated YAP/TAZ complex translocates to the nucleus to drive transcriptional activation (Figure 2). The phosphorylation/degradation strategy has been seen in many biological molecules for their turnover. For example, tumor suppressor p53 is subjected to Mdm2-mediated degradation in the cytoplasm, whereas phosphorylated p53 is stabilized in the nucleus.

MST1/2 in Hippo pathway can be activated without upstream kinases. The phosphorylation cascade is enhanced by MST1/2 dimerization (53). Active MST1/2 phosphorylates SAV1 and MOB1A/B (19, 29), which assists MST1/2 to recruit and phosphorylate LATS1/2 at their hydrophobic motifs (T1079 for LATS1 and T1041 for LATS2) (24, 54). Another key component in this action is NF2 (or Merlin), which directly interacts with LAST1/2 and promotes their phosphorylation (24). LATS1/2 subsequently undergoes autophosphorylation (18), and triggers the phosphorylation of YAP and TAZ for functional inactivation (55). Moreover, in parallel to MST1/2, two groups of MAP4Ks (mitogen-activated protein kinase kinase kinase kinase), MAP4K1/2/3/5 [homologs of *Drosophila happyhour* (Hppy)] and MAP4K4/6/7 [homologs of *Drosophila misshapen* (Msn)] directly phosphorylate LATS1/2 at their hydrophobic motifs and result in LATS1/2 activation, which consequently inactivates YAP/TAZ (23, 56, 57).

Overall, like many signaling pathways, the Hippo phosphorylation cascade is well-orchestrated and evolutionarily conserved. However, the ultimate outcome can be altered, either enhanced, or altered, by various signal stimulators. Conceivably, a single stimulator Wnt or growth factor, for example, may activate not only the Hippo pathway but also other molecular paths, thereby either toning down or escalating the outcomes. Nonetheless, there are multiple signal initiators for the Hippo pathway. The signal flow could be in either a concerted manner or ends up in chaos.

Among all the factors, how can those signals possibly work in a concert or contradictory manner? In short, GPCR either activates or inhibits the Hippo-YAP pathway depending on the signaling effected by the soluble Serum-borne lysophosphatidic acid and sphingosine 1-phosphophate (44). Soluble factor Amphiregulin binds EGFR and acts as an autocrine growth factor for establishing a positive autocrine regulatory feedback loop between EGFR and YAP1, which is important in cancer progression (37). Cell junction proteins Echinoid and E-cadherin inhibit YAP/TAZ activation. Echinoid physically binds and stabilizes the Hpo-binding partner Sav at adherens junctions. Loss of Echinoid compromises Yki phosphorylation, resulting in elevated Yki activity that increases Hpo-targeted gene expression and drives tissue overgrowth (39). Also, E-cadherin inhibits YAP/TAZ activation without involving the upstream signals of the Hippo pathway. This is achieved via the regulation of alpha/beta-catenin pathway (40).

## YAP and TAZ are WW Domain-Containing Proteins

The WW domain is a structural module that mediates protein-protein interactions through recognition of proline-rich peptide motifs (PRM) and phosphorylated serine/threonine-proline sites (58). WW domains are found in at least 52 different structural and signaling proteins in the human proteome. They participate in a variety of cellular processes and have been implicated in major human diseases such as cancer and neurodegeneration. In the Hippo pathway, SAV, KIBRA, YAP, and TAZ are the critical WW domain-containing proteins (59–61). While WW domains are responsible for protein/protein binding, the actual functional areas in the WW domain-containing proteins may not necessarily be on the WW domain areas. Phosphorylation in particular amino acid residues outside of the WW domain(s) may confer crucial molecular functions both *in vivo* and *in vitro*.

### The Configurations and Binding Motifs of YAP/TAZ

The human *YAP* gene is located on chromosome11q22. YAP protein was first identified as a proline-rich phosphoprotein, and is capable of binding the SH3 domain of Yes and Src protein tyrosine kinases (62). *YAP* mRNA is not only ubiquitously expressed in a broad range of tissues, but also is expressed during the entire developmental process (63). The human *TAZ* gene is mapped at chromosome 3q23-q24, and encodes TAZ protein (also known as WW-domain containing transcriptional regulator 1, WWTR1). Similarly, the *TAZ* gene is expressed in various tissues and amplified in many cancer cells (64).

**Figure 3:**
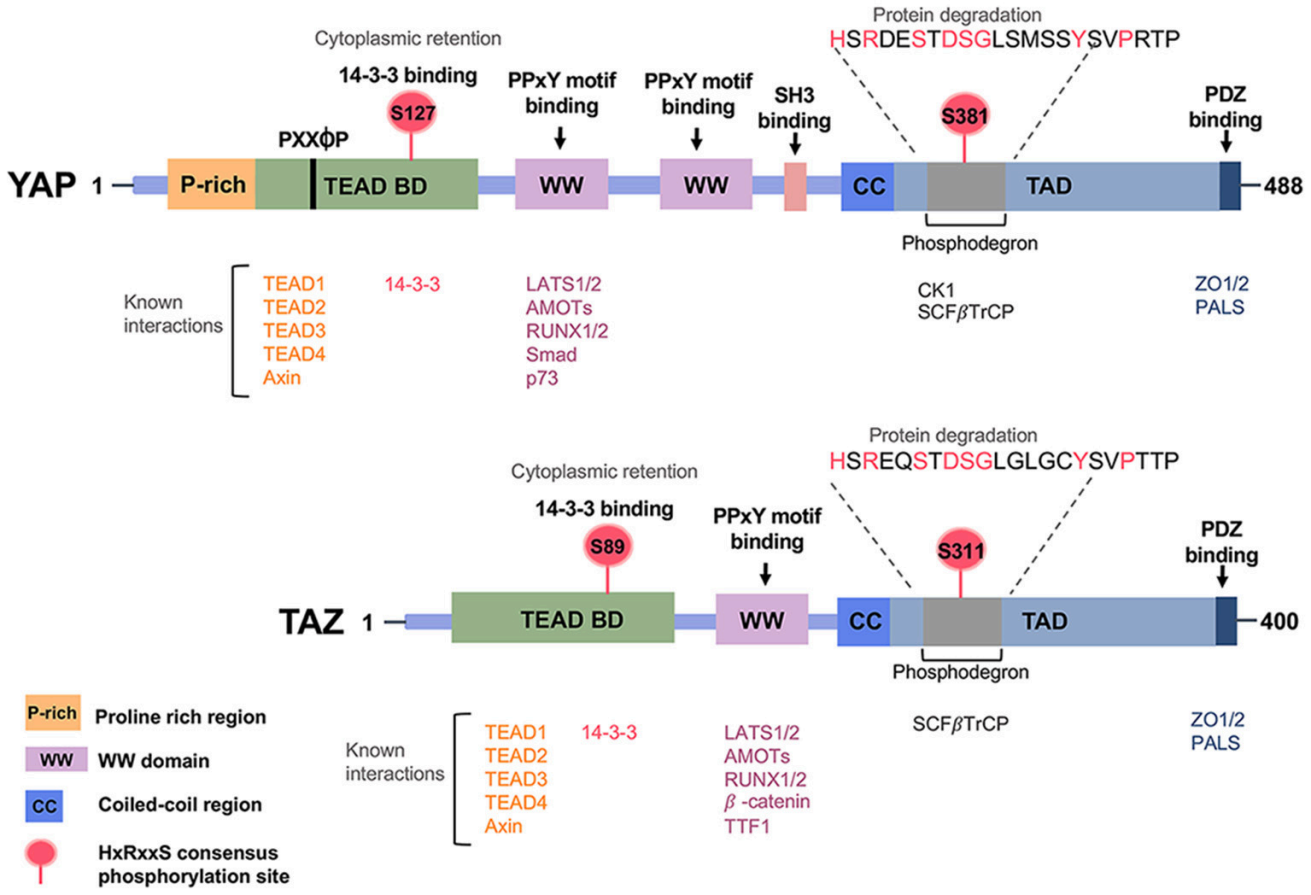
Regulatory domains of the Hippo pathway effector proteins YAP/TAZ. Prominent regions include the WW domain(s), the coiled-coil (CC) domain, the SH3-binding domain, the TEAD transcription factor-binding domain, the transcriptional activation domain (TAD) and the PDZ-binding motif.

Structurally, YAP and TAZ proteins share nearly half of the protein sequence identity and have a very similar topology. YAP protein has 488 amino acids, possessing a TEAD-binding region (TB), one or two WW domains in the isoforms, an SH3-binding motif, a coiled-coil domain, a transcription activation domain, an *N*-terminal proline-rich domain, and a *C*-terminal PDZ-binding motif. The WW domain has two-conserved tryptophan (W) residues separated by 20–23 amino acids. TAZ protein consists of 400 amino acids and has a similar domain organization with YAP, although it lacks the second WW domain, the SH3-binding motif, and the proline-rich domain (Figure 3).

The WW domains of YAP and TAZ physically interact with the “PPXY motifs (proline/proline/any amino acid/tyrosine)” in many proteins, including transcriptional factors (65). The TEAD-binding domain (TB) of YAP and TAZ recognizes the transcriptional enhancer factor domain family (TEAD) family of transcription factors and activates target gene expression, whereas the 14–3–3 binding motif is involved in the degradation of YAP and TAZ. As mentioned previously, in a canonical pathway, LATS phosphorylates YAP/TAZ and promotes the binding of 14-3-3 with YAP/TAZ. 14-3-3 binds YAP/TAZ via the phosphorylation sites S127 of YAP and S89 of TAZ, which results in cytoplasmic retention of YAP/TAZ. Certain upstream regulators, such as α-catenin, require the binding of 14-3-3 with the YAP pS127 site for subsequent inhibition of cell proliferation (66). Protein Phosphatase 2A (PP2A) dephosphorylates the 14-3-3 docking site on YAP, resulting in the nuclear import of YAP. The PDZ-binding motif is required for binding with another PDZ domain, and this can be found in many transmembrane or cytoskeleton-associated proteins. The PDZ-binding domains display to direct the cellular distribution of YAP and TAZ (67, 68). Functionally, YAP and TAZ are transcriptional co-activator and work synergistically with transcription factor companions such as TEAD and RUNX1/2, SMAD family members (69–71).

### YAP/TAZ Action in Downstream Gene Targets

YAP and TAZ cannot physically bind chromosomal DNA and thus act as co-activators with DNA-binding transcription factors to regulate a large number of target genes (72–75). Both proteins regulate the gene expression mainly by binding TEAD1-4 [homologs of *Drosophila* Scalloped (Sd)] (21). The interaction between YAP/TAZ and TEAD drives the expression of numerous genes for cell proliferation, survival, and migration. The gene products are the cell cycle regulator cyclin E, cell-death inhibitor Diap1, connective tissue growth factor (CTGF), cysteine-rich 61 (Cyr61), fibroblast growth factor (FGF1), and bantam microRNA (37, 69). Mouse models harboring the deletion of Mst1/2, Sav1, Mob1A/B, NF2, or Last1/2, or Yap overexpression, exhibit up-regulated *Tead* target gene expression, and increased expansion of progenitor cells and tissue overgrowth (76–79). thereby manifesting the importance of these genes in the Hippo pathway (Figure 2).

Importantly, Tondu-domain-containing growth inhibitor (Tgi) and Vestigial-like family member 4 (VGLL4, an ortholog of Tgi) are well known regulators of Yki and YAP/TAZ, respectively. Tgi directly competes with Yki for Sd binding in nucleus, restrains the Yki-regulated transcription (22, 69). When Hippo signaling cascade is on, Tgi and Sd form a complex to exert transcriptional repression. Conversely, when Hippo signaling is off, Yki translocates into the nucleus and takes the place of Tgi from Sd, permitting the expression of Yki target genes (69). By the same token, in mammals, VGLL4 competes with YAP/TAZ for TEAD binding (80, 81).

The YAP/TAZ complex also regulates the expression of certain genes via binding specific factors. For instance, the YAP/TAZ complex interacts with the nucleosome remodeling and histone deacetylase (NuRD) complex, and decreases the transcription of target genes (82). Moreover, YAP is identified as a regulator for miRNA biogenesis by modulating miRNA ‘processing enzymes microprocessor or Dicer complex (83, 84). Further, YAP directly induces the production of miR-130a, which represses VGLL4 expression, and forms a positive feedback loop for promoting the amplification of YAP activity (85). Hence, the observations suggest that YAP/TAZ regulate gene transcription through coordination of multiple mechanisms (86).

## Hippo Signaling in Organ Size Control and Tissue Homeostasis

The remarkable effect on organ size and tissue homeostasis regulation is the best-known feature of the Hippo pathway. How the Hippo pathway senses the physiological cues to modulate organ size is largely unknown. It is generally believed that the mechanical force or tension may determine the tissue growth and restrain YAP/TAZ activation when the organ reaches its proper size. Organ sizes are restricted/induced by some soluble factors via the autocrine/paracrine mechanism in a concentration-dependent manner (87). Still, a crucial point is that how the cells on the outer surface of an organ sense the overgrowth from the inner mass of the organ, and vice versa. Does the Hippo pathway coordinate the even cell growth, cell shape, and spatial relationship of all cells in an expanding organ?

### Effects of Hippo Signaling on Organ Size Regulation

In *Drosophila*, dysregulation of the Hippo pathway kinases (*Hpo* and *Wts*) or upstream regulators (*Ex, Mer, Kibra, Ft*, etc.) promotes Yki-induced cell proliferation and finally leads to tissue/organs overgrowth (16, 25, 26, 28). Under physiologic conditions, the liver returns to its normal size while Yap expression is restored at the background level (88). The long-term persistent Yap activation causes the onset of hepatocellular carcinoma (30). Liver conditional knockout of *Mst1/2, Sav1*, or *Nf2* causes liver enlargement (76, 89). Moreover, deletion of *Sav1, Mst1/2*, or *Lats1/2* gene brings upon embryonic heart enlargement.

The genetic manipulation of *Yap* robustly affects the proliferation and survival of cardiomyocytes (90–92). Substantial evidence revealed that tissue-specific deletion of *Yap* gives rises to abnormalities in several organs, including heart, skin, and kidney (93–95). Abnormal activation of YAP/TAZ makes aberrant influences in multiple organs, such as liver, heart, stomach, and spleen. However, tissue-specific deletion of *Yap* does not cause defects in the breast and intestine (78, 96). Mammary glands and intestine remain relatively normal upon *Yap* deletion (96–98). These findings imply that differential regulation of the Hippo pathway is utilized for size regulation among organs. Despite the outstanding findings, the mechanical force from within or outside the organ to balance the cellular size, shape, and tension, along with regulation by the Hippo pathway and/or others, is not well-defined.

### Hippo Signaling Regulation in Tissue Regeneration

For adult organisms, damage, and impair can barely be avoided during the lifetime. Thus, tissue regeneration is especially important. The Hippo signaling pathway, especially Yap, participates in organ regeneration. For example, liver regenerates efficiently post damage (e.g., partial hepatectomy), due to the proliferation of hepatocytes to restore liver mass. The transcriptional activity of Yap/Tead is increased during liver regeneration, meanwhile Mst1/2 and Lats1/2 are down-regulated toward the completion of tissue repair. When the regeneration is finished, the Yap activity is repressed and Mst1/2 and Lats1/2 inhibition is released automatically (89, 99–101).

Similarly, the study in the mouse model showed that intestines can effectively repair and regenerate from colitis induced by dextran sulfate sodium (DSS). However, the regenerative capability is severely obstructed upon tissue-specific knockout of *Yap* (96). Marvelously, overexpression of *Yap* restores some myocardial regenerative capability, although the regeneration of the adult heart is very limited. In contrast, specific deletion of *Yap* obstructs the regeneration of the neonatal heart (91, 92, 102). Again, the studies imply that organ formation and morphogenesis is not only just the proper function of YAP but also other cellular factors are involved.

### YAP/TAZ in Progenitor Cell

Progenitor cells play an important role in tissue homeostasis and regeneration. A high YAP/TAZ activity has been observed in the stem cells or progenitor cells of multiple tissues. Activation of YAP, which usually leads to the expansion of progenitor cells, impairs cell differentiation of target tissues such as intestine, liver, skin, and nervous system (96, 97, 103, 104). In intestinal crypts, YAP is highly localized in the nucleus promoting target gene expression in basal progenitor cells and in intestinal stem cells (30, 96, 105). In chick embryo, YAP and its transcriptional targets CTGF and CYR61, which are known to stimulate cell proliferation, are abundant during early development of the stomach (106). Moreover, YAP is highly expressed in the nuclei of single-layered basal epidermal progenitors. YAP is crucial in promoting the expansion of basal epidermal progenitors, proliferation, and inhibition of terminal differentiation through transcriptional target Cyr61. After initiation of hair follicle morphogenesis, YAP translocates to the cytoplasm of differentiating cells (107). Taken together, the capability of YAP/TAZ on cell proliferation and progenitor cell expansion suggests its decisive role in organ development, tissue homeostasis, and injury-induced regeneration. As a negative control mechanism, YAP/TAZ has to be under proper phosphorylation in order to be subject to degradation by the 14-3-3/SCFβTrCP/ubiquitin/proteasome system.

## Hippo Pathway in Early Embryonic Development, Stemness Maintenance and Promoting the Production of Induced Pluripotent Stem Cells (iPSCs)

Stem cells possess the unique properties of self-renewal and differentiation potency, which undergo asymmetric replication to divide one daughter cell identically to the parental cell and another daughter cell differentiating into destined cell type (108). In mammals, there are two major types of stem cells: pluripotent stem cells and somatic stem cells (109). Pluripotent stem cells, such as embryonic stem cells (ESCs), which are isolated from the inner cell mass (ICM) of blastocysts during development. These cells are capable of differentiating into near all cell types of three germ layers—ectoderm, endoderm, and mesoderm. Somatic stem cells are isolated in various adult tissues. The somatic stem cells and progenitor cells function in the repair system in different adult tissues and maintain tissue growth, homeostasis and regeneration through expanding cell numbers and replacing old or injured cells. Stem cells play critical roles in both embryonic developmental and adult stages. Therefore, it is important to understand the molecular mechanisms underlying the stemness maintenance. Appropriate tools are needed to pry into the perfect prospectus regarding how one single cell grows into a multicellular organism under a concerted and yet a regulatory signaling network.

### YAP/TAZ in TE and ICM Specification

Stem cells possess the drive to proliferate, differentiate, and migrate during development and tissue regeneration, in which the Hippo pathway is involved. The first cell lineage specification during embryonic development is a special feature of mammalian development with the emergence of the inner cell mass (ICM) and trophectoderm (TE).

**Figure 4:**
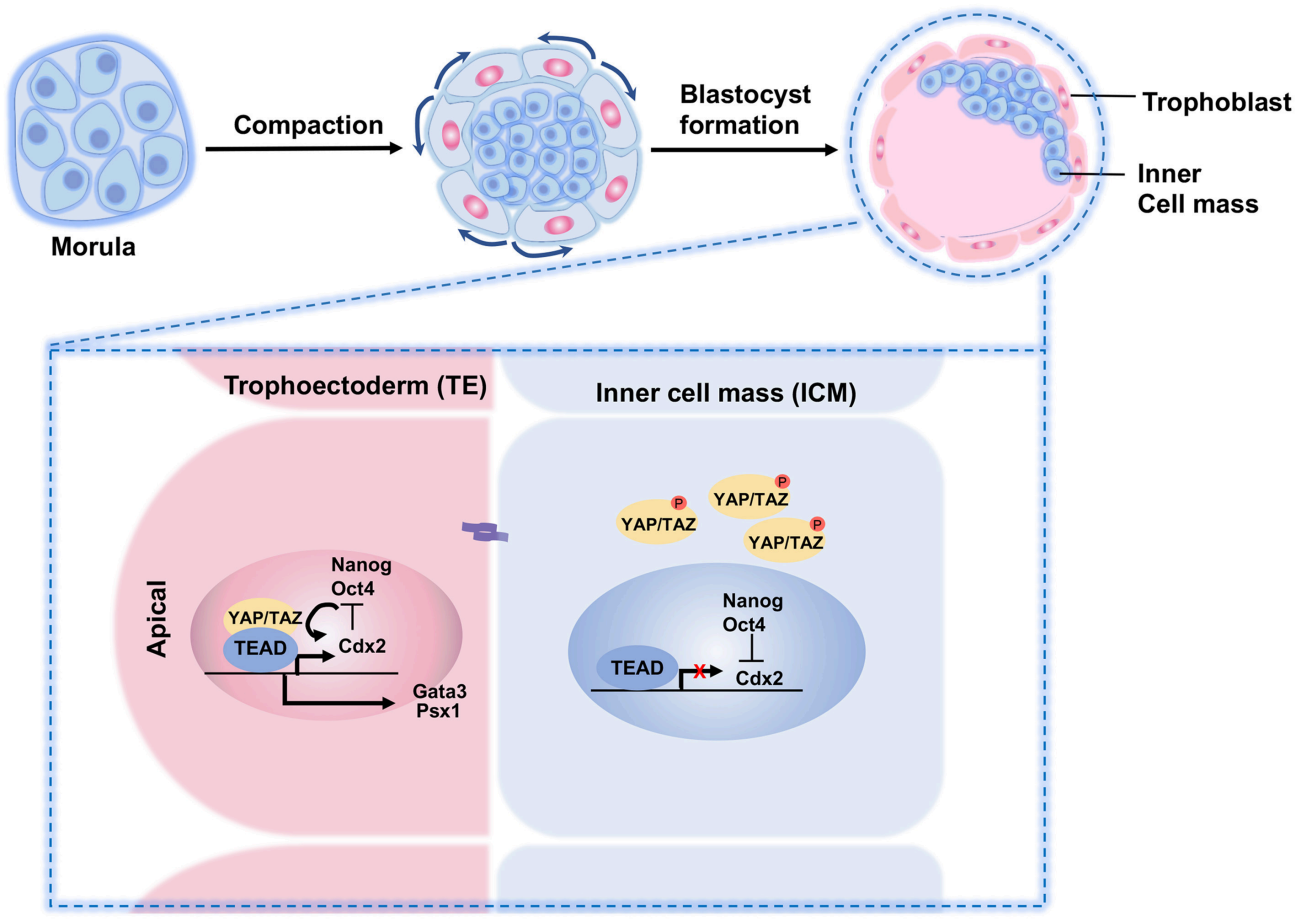
A schematic illustration of YAP/TAZ regulation of TE and ICM specification in preimplantation embryo. During preimplantation, the outer blastomeres of the embryo form an outer epithelial trophoectoderm (TE) that envelopes the remaining blastomere and the inner cell mass (ICM). The Hippo pathway plays a crucial role in the cell fate determination. In the outer cells, nuclear YAP and TEAD4 regulate specification of the TE lineage through activation of the TE-specific genes such as Cdx2 and Gata3. In the inner cells, cell-cell adhesions influence the Hippo signaling. Activated Hippo pathway impairs YAP nuclear localization in the ICM lineage, thereby limiting TEAD4 transcription and abrogating the expression of outer TE-specific genes.

In mice, after the gametes are fertilized in the oviduct, the embryo divides into an eight-cell morula with a relatively slow dividing rate. Each cell of the morula is called a blastomere. During the preimplantation stage, the blastomeres form adherens and tight junctions, and acquire apical-basal polarity, as well as an increase in the surface contact with its neighbors in a process called compaction (110). This creates the polarization of the cells within the morula. As these cells divide into the 16-cell stage, the innermost and more compacted cells lose their polarity. The differences between the inner and the outer cells bring about a disparate distribution of TAZ/YAP proteins, followed by further division to yield a blastocyst composed of about 32 cells. By entering the blastocyst stage, the subcellular localization of TAZ/YAP determines the first cell fate choice in the embryo—the settlement of embryonic cells to become either TE or ICM. For TE cells, TAZ/YAP is accumulated in the nuclei but distributed all over the cytoplasm of the ICM cells (111). The TE cells at the outer layer of the blastocyst form extraembryonic tissues, whereas ICM gives rise to the definitive structures of the fetus (112) (Figure 4).

### YAP/TAZ in Embryonic Development

*Yap* knockout mouse exhibits arrest in the division in the blastomere before entering the morula stage (16–32 cells) and embryonic lethal at E8.5 with a vascular defect of yolk sac (63, 111, 113–115). Similarly, deletion of both *Yap* and *Taz* results in cell fate specification defects, leading to embryo death at the morula stage prior to the specification of TE or ICM (87, 111), again suggesting the Hippo pathway and YAP/TAZ play vital roles at early embryonic development.

When the Hippo signaling pathway is inactive, YAP promotes trophectoderm fates in the embryo. However, when the Hippo signaling is activated, YAP relocates from the cell nucleus to the cytoplasm to promote pluripotent cell fates (116, 117). The differential distributions of YAP/TAZ in the TE and ICM causes distinct gene expression signatures, where the nuclear TAZ/YAP complex directs a TE-specific transcriptional program by interacting with TEAD transcription factors. For example, at the blastocyst stage, YAP/TAZ induces *Cdx2* and *Gata3* transcription by modulating the activity of TEAD in the outer TE cells (111, 118). High YAP activity is necessary for TE specification. For example, in *Tead4*^−/−^ embryos, all blastomeres are specified to the ICM lineage (119–121).

### TEADs

TEADs are the earliest genes highly expressed during embryo development, and are the transcription factors with YAP/TAZ as cofactors. Among them, TEAD4 is required for specification of the TE lineage during preimplantation in mammals. TEAD4 activity depends on the nuclear localization of YAP, which is regulated by cell-cell contacts and LATS1/2-mediated phosphorylation for negative regulation. Phosphorylated YAP undergoes degradation in the cytoplasm. Increased nuclear localization of TAZ/YAP, resulting from the deletion of *Lats1/Lats2*, leads to amplified *Cdx2* expression, which prevents proper specification of the ICM (111). In addition, the junction-associated angiomotin (AMOT) family proteins angiomotin (Amot) and angiomotin-like 2 (Amotl2) are essential for Hippo pathway activation and appropriate cell fate specification (27). Depleting LATS1/2, NF2, or AMOT/AMOTL2 turns all cells into TE, and these embryos fail to establish ICM-derived tissues (119–121). In contrast, knockout of TEAD leads to the loss of *Cdx2* expression and prohibits the embryos to form the TE with a consequence of all cells transforming into ICM (24, 122).

Depletion of both *Mob1a* and *Mob1b*, which are regulators of LATS1/2 activity, also results in developmental defects, with embryo arrest at E6.5 prior to gastrulation (110). Analysis of *MOB1A/B*-deficient blastocysts revealed aberrant nuclear YAP localization and modest growth failure of ICM, with few defects associated with the TE. Similar to the *Lats1/2*-deficient embryos, depletion of both *Amot* and *Amotl2* also increases nuclear YAP localization and *Cdx2* expression throughout both inner and outer cell populations, thereby resulting in severe pre-implantation defects. However, depletion of either *Amot* or *Amotl2* alone did not cause pre-implantation defects, indicating redundancy between AMOT family members (117).

Additionally, Neurofibromin 2 (NF2) (or Merlin) and AMOT, two upstream components of the Hippo pathway, facilitate YAP phosphorylation via LATS1/2 during cell fate specification of mouse preimplantation development. Mechanistic studies have revealed that LATS1/2 induces the phosphorylation of AMOT in the inner cells of the pre-implantation embryo, promoting its association with NF2 at cell membranes, and consequently amplifying the TAZ/YAP phosphorylation (117). Altogether, these results demonstrate a critical role for YAP and Hippo signaling cascade in the process of cell fate specification and development in early embryos.

### TAZ/YAP in Stem Cell Pluripotency

The Hippo pathway regulates pluripotent stem cells *in vitro*. TAZ/YAP participates in the differentiation of embryonic stem cells (ESCs) derived from the ICM of the blastocyst. ESCs are pluripotent stem cells capable of self-renew and differentiating into near all functional cell types in an adult organism. YAP is highly expressed in self-renewing ESCs. Downregulation of YAP protein and mRNA levels correlates with significantly decreased pluripotent markers along the line of ESC differentiation. In addition, YAP is sequestered and thereby inactivated in the cytoplasm during ESC differentiation, and consequently a large number of major genes for stem cell maintenance and function, including *Nanog, Oct3/4*, and *Sox2*, are repressed. Overexpression of *Yap* inhibits ESC differentiation and maintains stem cell properties even under differentiation culture conditions, while knockdown of *Yap*/*Taz* is sufficient to result in the impairment of ESC phenotype (123–126). Furthermore, restricting YAP and TEAD2 expression or inhibiting TEAD function induces differentiation toward the endoderm lineage. In contrast, deletion of *Mst1*/*2* in ESCs induces cell proliferation and breaks the process of differentiation (127). Further, bone morphogenetic protein (BMP) and leukemia inhibitory factor (LIF) signals maintain mouse ESCs in an undifferentiated and pluripotent state, whereas human ESCs require fibroblast growth factors (FGF), BMP, and transforming growth factor-β (TGF-β) to maintain pluripotency.

A fine-tune of growth factor-induced and cytoskeleton-associated cues are crucial in the maintenance and balance between differentiation and self-renewal in ESCs. These signals ultimately control the levels and actions of a core transcriptional circuitry consisting of OCT4 (also known as POU5F1), NANOG and SOX2 (128). YAP and TEAD2 activate the expression of ESC master transcriptional regulators OCT4 and NANOG in mammalian ESCs. Also, nuclear TAZ/YAP activity is required to integrate growth factor signals with these core transcriptional regulators to maintain the ESC pluripotent state. Human ESCs require signals induced by FGFs and members of the TGFβ family (129). TGFβ stimulates the action of serine/threonine kinase receptors that phosphorylate and activate the SMAD2/3 class of transcription factors (130). TAZ/YAP proteins form complexes with phosphorylated SMAD2/3 (123, 126). In the nucleus, the TAZ/YAP-SMAD2/3 complexes bind TEAD transcription factors, and the core stem cell regulator OCT4, and together mediate the pluripotent state (124). Mechanistically, this complex assembles with factors that make up the nucleosome remodeling and deacetylation (NuRD) complex to buffer the expression of pluripotency genes and repress genes that define mesoendoderm. Upon mesoendoderm specification, the TAZ/YAP-TEAD-OCT4 complex dissociates from the SMADs, allowing the SMADs to activate the forkhead transcription factor FOXH1 and drive differentiation (124).

Similarly, mouse ESCs also require precise *Yap* levels to maintain their pluripotent state. Knockdown of *Yap* in mouse ESCs leads to loss of OCT4 and SOX2 expression, and consequent differentiation (125).

YAP may induce the expression of pluripotency-associated genes, which promote ESC self-renewal. It is suggested that YAP is dispensable for self-renewal but is required for the differentiation of ESCs. Knockdown or knockout of *Yap* does not alter ESC self-renewal but impairs their differentiation (131, 132). TAZ and the TEADs are also dispensable for ESC self-renewal (132). Deletion of *Lats2* in ESCs impairs their pluripotency and ability to differentiate (133). The discrepancy among these studies is likely due to the presence of unknown factors, which affect the Hippo pathway in different cell lines, as well as variations in cell culture and experiments.

### YAP in Induced Pluripotent Stem Cells (iPSCs)

The role of YAP in controlling pluripotency is seen in induced pluripotent stem cells (iPSCs), which was established by using the ectopic expression of *Oct3/4, Sox2, Klf4*, and *c-Myc* genes closely resemble that of ESCs in phenotype and differentiation potential (124, 134). YAP is activated during the reprogramming process of human embryonic fibroblasts into iPSCs, and the addition of YAP to original four factors increases the reprogramming efficiency in mouse embryonic fibroblasts, further confirming a positive role of YAP in stemness (135). As in human ESCs, the interaction between YAP and TEADs directs transcriptional circumstances important for maintaining pluripotency. Ectopic expression of a nuclear-localized mutant YAP promotes mouse ESC self-renewal and increases the efficiency of iPSC reprogramming (125). In human iPSC, knockdown of *LATS2* increases the reprogramming efficiency of iPSCs (136). Moreover, a recent study reported the success in reprograming of iPSCs derived from primary human amniotic epithelial cells (HuAECs) through the introduction of OSY (*OCT3/4, SOX2*, and *YAP*) to activate the Hippo-Yap signaling pathway (135).

While the nuclear TAZ/YAP complex exhibits crucial roles in ESCs cultured *in vitro*, studies using mouse blastocysts indicate that TAZ/YAP is in the cytoplasm of the ICM, the region from which ESCs are derived. The observations suggest that pluripotent ESCs exist very transiently *in vivo*, and that changes in TAZ/YAP localization might provide a mechanism to integrate microenvironmental cues during cell differentiation. Indeed, a mechanical microenvironment dramatically affects stem cell fate (137).

## Hippo Signaling in Tumorigenesis and Tumor Initiating cells

The Hippo pathway is critical in homeostasis, and is utilized by cancer cells for growth and progression. Mechanically, cytosolic phosphorylated YAP/TAZ inhibits tumor growth. Upon de-phosphorylation, YAP/TAZ undergoes nuclear accumulation and promotes cell and tumor growth. In mouse, long-term YAP activation results in liver cell transformation and tumor development (138). The oncogenic activity of YAP is highly dependent on TEAD-mediated gene transcription. Dominant negative TEAD inhibits YAP-induced liver cancer by sequestering YAP and TAZ in the cytoplasm (138). Clearly, these findings suggest that the action of YAP/TAZ fails to distinguish what is a normal or an abnormal cellular event, and there is no apparent regulatory mechanism in the nucleus to override the action of YAP/TAZ.

### YAP/TAZ Gene Alterations and Protein Fusions

Upregulated expression or nuclear accumulation of YAP/TAZ has been observed in various types of human cancers, including liver, breast, lung, colon, ovary (139–141). Gene alteration and fusion genes involving *YAP* and *TAZ* have also been examined in human cancers. For example, *NF2* mutation causes neurofibromatosis 2, schwannomas, meningiomas, and mesothelioma (142). Heterozygous deletion of Yap completely blocks liver tumorigenesis in *Nrf2* knockout mice (47). Statistics with clinical epithelioid hemangioendotheliomas specimens revealed that almost all cases carry *TAZ*-*CAMTA1, TAZ*-*FOSB*, or *YAP*-*TFE3* fusion genes (101, 143–147). Besides, in a portion of ependymal tumors, *YAP* gene is found to fuse with MAMLD1 or *C11orf95* (145, 148).

A similar finding is also observed in neuroblastoma, neural stem cells carrying the *YAP*-*C11orf95* fusion gene tent to effectively form brain tumors when transplanted into mice (148). In addition, a familial *YAP* point mutation (R331W) has also been reported to correlate with a high incidence of lung adenocarcinomas (149). Although in these fusion genes, YAP/TAZ proteins are not unabridged, all YAP/TAZ fusions proteins preserve their *N*-terminal TEAD binding domain. This indicates that the fusion proteins still have the binding ability to TEAD and are able to activate the TEAD-dependent transcriptional program for promoting tumorigenesis. Aberrant mutations of components within the Hippo pathway affect the function of YAP/TAZ leading to oncogenesis. *LATS1/2* mutations or gene fusion, which may cause YAP/TAZ activation, have also been sporadically identified in different cancers (150–154). Evidence of the Hippo pathway in tumorigenesis based on mouse models is summarized in Table 2. In addition, crosstalk with other cancer-related signaling pathways, such as *KRAS, APC*, and *LKB1* mutations, have all been reported to contribute to upregulate the YAP/TAZ activity in cancers (156–158).

**Table 2:** Genetic alteration of Hippo pathway in human cancer.

**Gene**	**Alteration**	**Cancer type**	**References**
*NF2*	Mutation or deletion	Mesothelioma Neurofibromatosis type 2 Schwannoma Meningioma	(142)
*LATS1/2*	Gene Fusion (*LATS1-PSEN1*)	Mesothelioma	(154)
	*LATS2* deletion	Mesothelioma	(152)
	*LATS2* mutation	Sporadic in different cancers	(151)
*YAP*	Amplification	Hepatocellular carcinoma Medulloblastoma Esophageal squamous cell carcinoma	(153)
	Mutation (R331W)	Lung adenocarcinoma	(149)
	Gene Fusion (*YAP-TFE3, YAP-ESR1, YAP-C11orf95*, and *YAP-MAMLD1*)	Epithelioid Hemangioendothelioma Luminal breast cancer Ependymal tumors	(146) (144) (147) (145) (148)
	Deletion	Hematological cancer	(155)
*TAZ*	Gene Fusion (*TAZ-CAMTA1*, and *TAZ-FOSB*)	Epithelioid hemangioendothelioma	(143) (144) (101)

### YAP/TAZ in Cancer Metastasis

Cancer metastasis is responsible for a huge portion of cancer-associated deaths. It has been reported that high YAP or TAZ activity enables the cells to escape contact inhibition and anoikis, and support anchorage-independent growth (55, 156, 159). YAP or TAZ activation promotes metastasis by influencing many of processes related to metastasis, such as epithelial-to-mesenchymal transition (EMT), invasion, extravasation, and escaping from the immune system. Substantial evidence indicates that in addition to promoting tumor growth, YAP activation is sufficient to drive cancer metastasis (160). For example, YAP promotes tumor metastasis through inducing the expression of Zinc finger E-box-binding homeobox 1 and 2 (ZEB1/2) for stimulating EMT (161). Activation of YAP through the loss of leukemia inhibitory factor receptor (LIFR) promotes metastatic colonization of breast cancer cells (162, 163). In fact, YAP/TAZ and TEADs in metastasis of numerous cancer types have also been implied, including melanoma (162), lung cancer (164–166) breast cancer (163, 167–169), cholangiocarcinoma (170), gastric cancer (171, 172), ovarian cancer (173), colorectal cancer (174, 175). Clinical data showed that YAP or TAZ expression or nuclear localization is increased in metastatic tumors compared to primary tumors in pancreatic cancer (176), breast cancer (177, 178), and prostate cancer (179).

### YAP/TAZ in Drug Resistance and Tumor Relapse

In addition to metastasis, drug resistance, and tumor relapse are the hard cracking nuts for cancer therapy. YAP/TAZ activity also correlates with drug resistance and cancer recurrence. One study has analyzed the gene expression profile of several cancer cell lines, and uncovered the immune checkpoint molecule PD-L1 as a target of Hippo signaling (180). In this process, PD-L1 expression is suppressed by the upstream kinases MST1/2 and LATS1/2 of the Hippo pathway, while TAZ and YAP enhance PD-L1 levels in breast and lung cancer cell lines. Critically, TAZ-induced PD-L1 upregulation in human cancer cells is sufficient to inhibit T cell function (181). Further, *in vitro* studies indicate that cultured breast cancer cells exhibit high YAP/TAZ activity and confer drug-resistance to many routinely used chemotherapeutic drugs, such as taxol, 5-fluorouracil, and doxorubicin (182–184). Lung and colon cancer cells with high YAP activity exhibit resistance to RAF- and MEK-targeted therapies (185). Furthermore, tamoxifen is one of the commonly used chemo-drug for the treatment of estrogen receptor (ER)-positive breast cancer. Recent study has shown that tamoxifen may activate YAP/TAZ by stimulating the membrane estrogen receptor GPER, which may explain why certain ER-positive breast cancers are insensitive to tamoxifen (186). Elevated gene expression of *YAP* is associated with cancer relapse in *KRAS*-driven colon and pancreatic cancers (155, 187). Taken together, inhibition of YAP/TAZ not only restrains tumorigenesis and tumor progression, but also has the potential to sensitize tumor cells to drugs in chemotherapies or target therapies and prevent cancer recurrence.

### Tumor Initiating Cells and the Hippo Pathway

A special subpopulation of highly tumorigenic cells, known as tumor initiating cells (TICs), has been proposed to harbor unique properties such as self-renewal and tumor-initiating potential. TICs are believed to be responsible for drug resistance, metastasis, recurrence, and major causes of cancer death (188). Chemotherapy and target therapy are currently the major treatments for patients with cancer. Unfortunately, these conventional therapies often fail to eradicate TICs, thereby permitting TIC-mediated cancer relapse. The YAP/TAZ complex has been shown to be a key regulator of TICs in various cancer types.

Tumor cells with a high YAP/TAZ activity display resistance to chemotherapeutics. YAP activation promotes the transformation of prostate epithelial cells to become an androgen-insensitive state and castration resistance *in vivo* (189). YAP facilitates the dedifferentiation and expands undifferentiated stem/progenitor cells, e.g., transformation of the mature hepatocytes into progenitor cells (89). YAP/TAZ activation leading to induction of TIC properties has been observed in multiple human cancers. In breast cancer, YAP occupies mammary stem cell signature gene promoters to induce breast TICs (190). TAZ expression is enriched in breast TICs with CD44^high^/CD24^low^ phenotype, which is required to sustain self-renewal and tumor-initiating properties (191). In addition, glucocorticoid hormone-induced YAP activation expands chemo-resistant breast TICs.

YAP is one of the major inducers of TIC properties, which directly upregulates the SOX9 transcription factor for controlling the differentiation of many cell types (190). Similarly, in osteosarcoma and glioblastoma, SOX2 promotes YAP activation to maintain TICs capacity (192). Glucocorticoid hormone-induced YAP activation induces expansion of chemo-resistance in breast TICs (191). In clinical specimens of urothelial carcinoma of bladder (UCB), the expression of YAP positively correlates with the expression of SOX2 and COX2. The tumor cells draw support from the proinflammatory role of the COX2/PGE2 pathway, and the growth-regulatory YAP recruits the stem cell factor SOX2 in expanding and sustaining the accumulation of TICs. In mouse xenograft study, activation of the COX2/PGE2 and YAP pathways promote acquired resistance to EGFR inhibitors (193). Taken together, YAP/TAZ plays an important role in tumorigenesis, cancer progression, and TICs maintenance. TICs are highly related to tumor spread, drug-resistance, and cancer relapse, and are responsible for treatment failure and poor prognosis. TIC-specific regulatory mechanisms of YAP/TAZ and Hippo pathway remain further elucidation.

## Targeting the Hippo Signaling Pathway for Therapy

**Figure 5:**
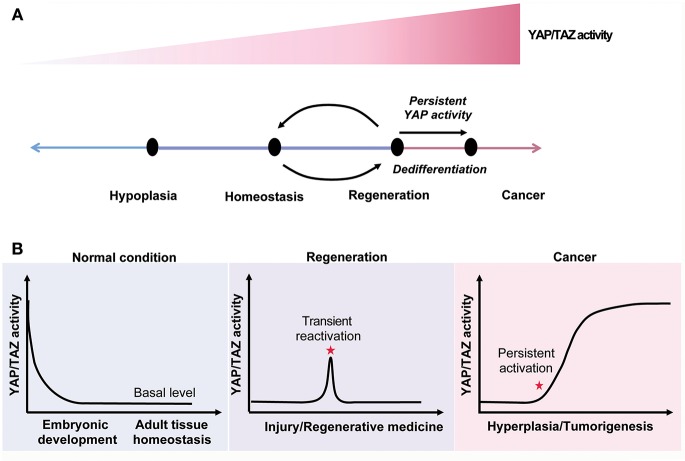
The Hippo signaling in tissue homeostasis, regeneration and cancer. **(A)** YAP/TAZ activity regulates tissue homeostasis and modulates the cell state. **(B)** YAP/TAZ activity is declined to a relative low level after birth. The YAP/TAZ activity is reactivated after tissue injuries and constitutively upregulated in cancer.

Nowadays, cancer becomes one of the notorious leading causes of mortality globally. A plenty of studies indicate that the Hippo pathway plays a key role in tumorigenesis. Aberrant Hippo pathway activity promotes cancer cells growth and leads cancer cells to acquire drug resistance (183). YAP and TAZ activity is often upregulated in cancer cells, but their activity is typically silent in normal resting tissue, suggesting that systemic YAP/TAZ inhibition allows cancer treatment without causing significant side effects (30, 117) (Figure 5).

### Small Molecular Drugs

Small molecular drugs are intended to disrupt the YAP/TAZ-TEAD complex and block the complex-mediated gene transcription. Verteporfin, an FDA-approved photosensitizer of porphyrin family molecules for treating macular degeneration, is currently the most widely used YAP inhibitor in research laboratories (138). Verteporfin binds YAP and disrupts the binding of YAP/TAZ with TEADs, and, alternatively, upregulates 14-3-3 expression to facilitate YAP/TAZ degradation (194–196). Verteporfin suppresses liver overgrowth induced by YAP overexpression or NF2 inactivation in mice (138). However, YAP-independent cytotoxic effect of Verteporfin has been reported (197–199). Moreover, pharmacological modulation of signal transduction pathways that crosstalk with the Hippo pathway or inhibition of YAP/TAZ target genes by combination therapy may provide promising approaches to target YAP/TAZ activity. Together, the potential of YAP/TAZ-TEAD as a therapeutic target may improve the current treatment strategies (200–202).

### Boosting the Hippo Pathway

In stark contrast, given the fact that the Hippo pathway is essential for organ development and regeneration, manipulating the components of the Hippo pathway would enhance drug design for regenerative medicine. Regenerative medicine refers to medical approaches to promote functional repair and regeneration of damaged tissues or organs, the practices can be separated into two major parts to reach the goal. One is stimulation of intrinsic repair process by molecular therapy, and the other is transplantation of tissues or stem/progenitor cells cultured in laboratories. Clinical doctors always face the hard situation with the shortage of donors compared to the increasing needs of tissue/organ transplantation. Thus, development of novel regenerative therapy is imperative.

YAP/TAZ activity is generally high during embryonic development, but soon declines to a basal level after birth. Upon tissue injury, YAP/TAZ activity is immediately reactivated in a transient manner. Transient activation of YAP/TAZ promotes the expansion of progenitors or dedifferentiation of mature cells to facilitate tissue regeneration. Thus, activation of YAP/TAZ is a potential strategy to promote tissue regeneration (Figure 5).

Inhibition of upstream kinases, such as MAP4K4, MST1/2, or LATS1/2, in the Hippo pathway, represents an ideal approach to induce YAP/TAZ activity and facilitate the process of tissue repair or regeneration, and possibly for treating degenerative diseases. Systematic or local delivery of Hippo pathway kinase inhibitors can induce this regenerative program. Recently, an MST1/2 inhibitor has been discovered and exhibits a good response in promoting liver and intestinal regeneration (203). Gene therapy is also an effective strategy (204), either by regulating YAP by the viral or viral-free system or introducing siRNA or miRNA for tissue regeneration (205).

Transplantation of *in vitro* expanded progenitors, organoids, or tissues are feasible methods. In recent years, a variety of organoids have been generated successfully *in vitro*, including stomach, liver, kidney, lung, intestine, brain, and retina (206). Nonetheless, it is a puzzle to command the complicated biological parameters, including cell types, organization, and microenvironment within an organoid system (207). Supporting evidence revealed the outstanding effects of YAP/TAZ transient activation in dedifferentiating mammary, neuronal, and pancreatic cells into a progenitor state, and these cells can be used to generate organoids for transplantation (208). Thus, modulating the Hippo pathway may represent a useful strategy for enrichment of progenitor cells or differentiated organoids for regenerative medicine.

In conclusion, the marvelous regulation of the Hippo pathway and YAP/TAZ in progenitor expansion and cell differentiation is forceful and feasible for promoting tissue regeneration. The Hippo pathway is generally considered as an effector in tumor suppression, and many anticancer drugs been developed (209). In facilitating regenerative medicine, small molecular drugs for Hippo activation can be implemented.

## Is WW Domain an All-Time Party Coordinator?

### All-Time Party Coordinator?

The WW domain is a well-known structural module that is responsible for protein/protein binding interactions (210, 211). The WW domain has three anti-parallel beta-sheets, which appears to facilitate protein/protein binding. In the human proteome, there are at least 52 WW domain-containing proteins, and more than 10,000 among all species. Each protein may have one or up to 4 repeats of WW domains in the amino acid sequence. The WW domain binds a proline-rich motif such as PPxY or PPPY in a target protein. Phosphorylation in specific residues within the WW domain enhances its binding capability as seen in WWOX (210, 211). In the Hippo pathway (212), there are 4 WW domain-containing proteins, namely SAV, KIBRA, YAP, and TAZ. Amazingly, YAP, TAZ, and/or the YAP/TAZ complex binds approximately 20 proteins, including p73, RUNXs, SMADs, PRGP2, Pax3, ErbB4, ASPP2, AMOTs, Wbp2, β-catenin, Parafibromin, RORα, and SET1A (212). Without a doubt, WW domain can be considered as an all-time party coordinator. Each WW domain-containing protein interacts with many proteins. The binding signals biological events for cell survival, death, and others. However, how can a single cell handle so many downstream signaling targets from an upstream stimulator during a single signaling run? And, this may end up with desired outcomes. Presumably, a specific downstream effector(s) stands out to solely carry out the signaling mission and exhibits the results, and meanwhile eliminates the outcome from other signaling co-effectors.

The signaling branches derived from the upstream signaling could exhibit conflicts among the downstream effectors. For example, Hippo pathway has a close connection with SMAD signaling (213). YAP enhances pSmad1-dependent gene transcription. In contrast, as a self-control mechanism, YAP promotes Smad7-mediated inhibition of TGF-β signaling (213). Nonetheless, there is a converge among Wnt, TGF-β, and YAP/TAZ pathway (214, 215). YAP takes the center of the signaling stage by participating in the gene transcription in the nucleus among these pathways (216).

### Tumor Suppressor WWOX in the TGF-β, Hyal-2, and Wnt Signaling That Cross Talks With the Hippo Pathway

**Figure 6:**
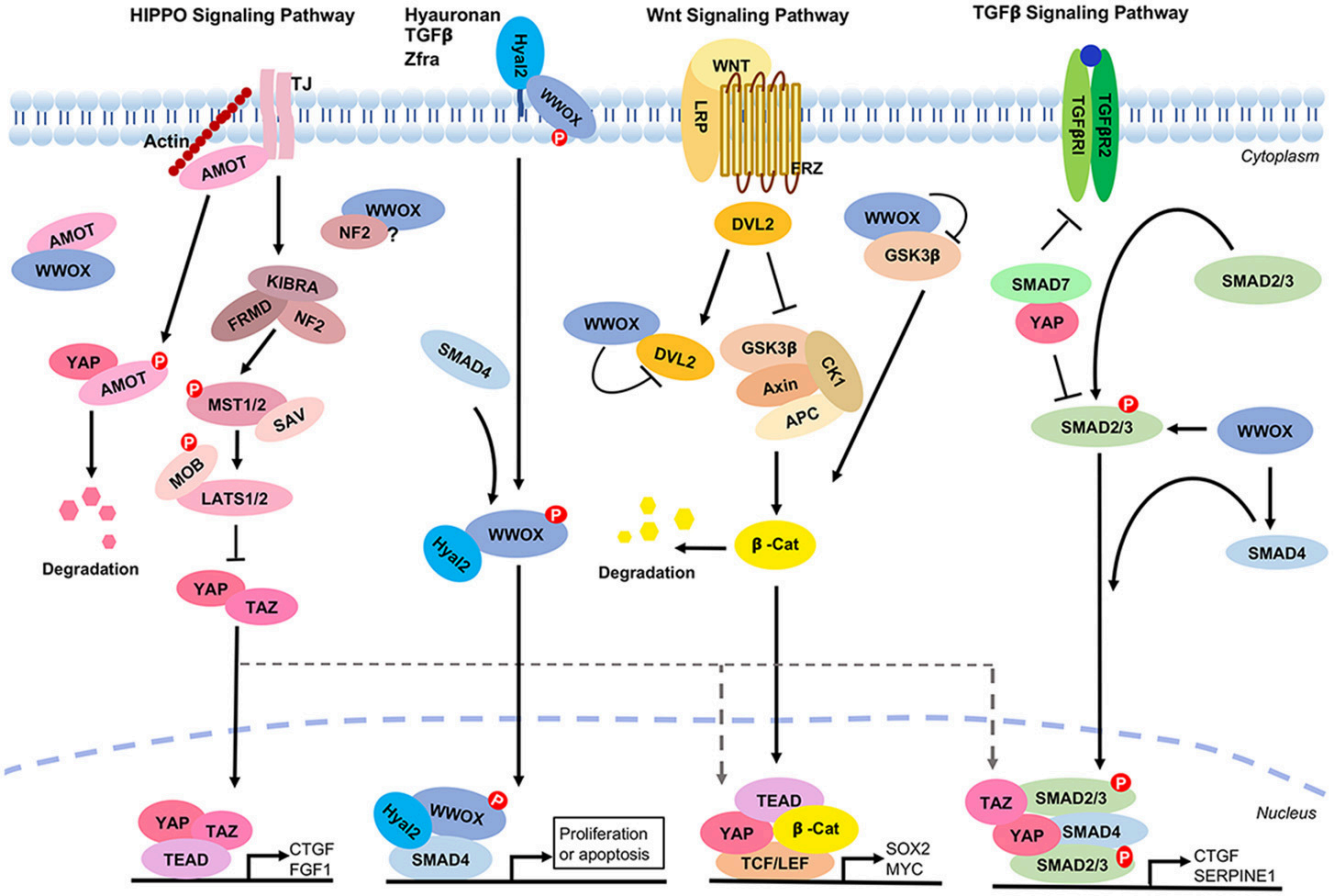
WWOX controls TGF-β, Hyal-2, and Wnt signaling pathways that cross talks with the Hippo signaling. A panel is shown for the Hippo, Hyal-2/WWOX/Smad4, Wnt and TGF-β signaling pathways. WWOX supports the Hyal-2 and TGF-β signal pathways. However, WWOX blocks the Wnt pathway by binding GSK3β and DvL2. WWOX binds AMOT and probably NF2 in the Hippo pathway, whereas the functional consequence of this binding is unknown.

Tumor suppressor WWOX is a potent inhibitor of the Wnt/β-catenin signaling via binding with Disheveled Dvl-2 to enforce the cytoplasmic retention of Dvl-2 and increased degradation of β-catenin (210, 217). WWOX binds Smad2, 3, and 4 in the TGF-β/Smad and Hyal-2 signal pathways to modulate cell proliferation or death (218–220) (Figure 6). Hyaluronan, TGF-β and Zfra (zinc finger-like protein that regulates apoptosis) utilize the Hyal-2/WWOX/Smad4 pathway (218, 219). Activation of the Wnt/β-catenin signaling allows nuclear translocation of TAZ to carry out gene transcription with TEAD. WWOX intervenes the event by directly binding and blocking the function of GSK-3β in the upstream complex of APC/AXIN/β-catenin/GSK-3β/YAP (221) (Figure 6). Indeed, YAP is involved in the transcription stage of the TGF-β, Wnt/β-catenin and Hippo signaling (222). WWOX controls the aforementioned signaling from the upstream steps and thereby affects the function of YAP in the downstream. Indeed, WWOX binds AMOT and probably NF2 in the upstream of the Hippo pathway.

### Protein Isoforms

WW-domain isoforms could be another issue. Some proteins have one WW domain, and others have more than one. YAP1 has two WW domain and YAP2 has one. YAP1 has up to 9 isoforms according to the NCBI protein database. For a single protein possessing more than one WW domains in the amino acid sequence, these WW domains may act in a concerted manner to boost the protein function (223). However, during the Hippo signaling, it is not clear which YAP isoform is the key executor to generate the intended outcome in a specific cell. The complexity of the signaling events can be observed not only in the Hippo signaling pathway but also in many signaling pathways (e.g., TGF-β, MAPK/ERK, and others). Among these pathways, there are crosstalk between each other. Knockdown of a single protein could generate a desired phenotype. However, the observation could be a sum of many signaling pathways.

### Concluding Remarks and Outstanding Questions

In summary, we have addressed the role of Hippo pathway in the regulation of embryonic development, organ growth, tissue regeneration, stem cell pluripotency, and tumorigenesis. As detailed in the above section, our concerns mainly point to the pathway-regulated biological effects and ultimate outcomes. Several outstanding questions are listed in the Table 3. Our hope is that more sophisticated tools are needed to predict and solve problems associated with multiple signal stimulators, crosstalk among the signal proteins in the pathway with others, functional efficacy of protein isoforms in the signaling, and problem-solving associated with conflict signaling.

**Table 3:** Outstanding questions.

**Gene and proteins**	
Gene and protein isoforms	What are the tissue-specific gene products from alternative splicing for the Hippo pathway? The Hippo pathway is evolutionarily conserved. Why do these proteins in the pathway readily respond to diversified stimulators? What are the key purposes for the biological outcomes? Do proteins isoforms compete with each other during the Hippo signaling? How can each cell handle the diversified signals? Signal integration or elimination?
WW domain proteins	What are the functional differences between two-WW-domain YAP1 and one-WW-domain YAP2? Further, YAP1 has 4 isoforms due to alternative splicing in the TAD domain. What are the functional differences among these proteins in terms of regulation of gene expression? Can which isoform act as a decoy protein to block the signal flow? KIBRA participates in both the Wnt/Hedgehog/Notch and Hippo signaling pathways. Which pathway does KIBRA have the most effect on the biological outcome? KIBRA binds LATS1 and LATS2. Which complex KIBRA/LATS1 or KIBRA/LATS2 is more effective in blocking YAP/TAZ? SAV1 binds MST1 and promotes apoptosis. How does apoptosis get activated?
Upstream of the Hippo signal pathway	Which upstream signaling molecules act in concert to suppress the YAP/TAZ activity, and which are in antagonistic manners? Which stimulator can override the signaling from others? Since the pathway is sensitive to diversified stimulators, are there unidentified agonists?
The Hippo signal flow	How many signal proteins in the Hippo pathway readily crosstalk with proteins from Wnt, TGF-β, and ERK pathways? Tumor suppressor WWOX interacts with these pathways. Can WWOX act as a modulator for optimizing the signaling outcomes? Lacking a real-time mode for cellular signaling pathways is hard to unravel the hidden events (e.g., promoter activation, protein/protein interactions, and etc.). For a signaling with so many branches in the downstream, or a signaling with many upstream stimulators, is there a dominant signal flow?

## Author Contributions

Y-AC and C-YL carried out the literature review, prepared schematic graphs, and wrote the manuscript. T-YC participated in drafting and prepared high-resolution figures and organized the references section. N-SC, S-HP, and H-FC provided advisory points to the first and other authors. N-SC thoroughly revised added challenging concepts and outstanding questions, developed new ideas, and completed the final version of the manuscript. All authors read and approved the final version.

### Conflict of Interest Statement

The authors declare that the research was conducted in the absence of any commercial or financial relationships that could be construed as a potential conflict of interest.

## Abbreviations

YAP: Yes-associated protein
TAZ: Transcriptional co-activator with PDZ binding motif
iPSCs: Induced pluripotent stem cells
Oct3/4: Octamer-binding transcription factor 4
Sox2: SRY (sex determining region Y)-box 2
PY motif: Proline-tyrosine motif
MST1/2: Mammalian sterile 20-like 1/2
SAV1: Salvador
LATS1/2: Large tumor suppressor homolog 1/2
MOB1A/B: MOB kinase activator 1A/B
TEAD: Transcriptional enhancer factor domain
VGLL4: Vestigial-like family member 4
NF2: Neurofibromin 2/ Merlin
MAP4Ks: Mitogen-activated protein kinase kinase kinase kinase
PRM: Proline-rich peptide motifs
TB: TEAD-binding region
PPXY: Proline/proline/any amino acid/tyrosine
PP2A: Protein Phosphatase 2A
Tgi: Tondu-domain-containing growth inhibitor
VGLL4: Vestigial-like family member 4
CTGF: Connective tissue growth factor
Cyr61: Cysteine-rich 61
TE: Trophectoderm
ICM: Inner cell mass
AMOT: Angiomotin
ESCs: Embryonic stem cells
BMP: Bone Morphogenetic Protein
TICs: Tumor initiating cells.
